# Milling itineraries dataset for a collection of crop and wood by-products and granulometric properties of the resulting powders

**DOI:** 10.1016/j.dib.2020.106430

**Published:** 2020-10-23

**Authors:** Charlène Fabre, Patrice Buche, Xavier Rouau, Claire Mayer-Laigle

**Affiliations:** IATE Planet, University of Montpellier - INRAE, 2 place Pierre Viala F-34060 Montpellier Cedex, France

**Keywords:** Agriculture by-products, Forestry by-products, Dry milling, Biomass powders, Laser granulometry, Particle size distribution, Specific surface area, Comminution

## Abstract

Lignocellulosic biomass represents a readily available reservoir of functional elements that can be an alternative to fossil resources for energy, chemicals and materials production. However, comminution of lignocellulosic biomass into fine particles is required to reveal its functionalities, improve its reactivity and allow practical implementation in the downstream processing steps (carrying, dosage, mixing, formulation, shaping…). The sources of lignocellulosics are diverse, with two main families, being agricultural and forest by-products. Due to plant specificity and natural variability, the itineraries of particle size reduction by dry processing, the behavior upon milling and therefore the characteristics of resulting powders can deeply vary according to various raw biomasses [[1], [2]].

This data article contains milling itineraries and granulometric properties of the resulting powders obtained from a collection of by-products from crops (flax fibers, hemp core, rice husk, wheat straw) and woods (pine wood pellets, pine bark, pine sawdust, Douglas shavings, chestnut tree sawdust) representative of currently used lignocellulosic biomass. Samples provided in the form of large pieces (hemp core, pine bark, Douglas shavings) were successively milled using different mills to progressively reduce the matter into coarse, intermediate and finally fine powders. The other samples, supplied as sufficiently small format, were directly processed in the fine powder mill. The machine characteristics and their operating parameters were recorded. The granulometric properties of the powders were analyzed with a laser granulometer and the main indicators related to the particle size distribution (PSD) are presented: (i) d10, d50 (or median diameter) and d90 which are the 10th, 50th and 90th percentiles of the cumulative volume distribution; (ii) the span, which evaluates the width of the particle size distribution; (iii) the calculated specific surface area of the powders which represents the sum of total surface exhibited by the particles per unit of gram and for some powders. The whole particle size distribution of a subset of produced powder samples are also provided for different milling times to illustrate the kinetics of particle size reduction.

These data are stored in INRAE public repository and have been structured using BIOREFINERY ontology [3]. These data are also replicated in atWeb data warehouse providing additional query tools [[3], [4]].

## Specifications Table

**Table:** 

Subject	Biomaterials
Specific subject area	*Milling itineraries and granulometric properties of resulting powders for a collection of crop/wood byproducts*
Type of data	*Table*
How data were acquired	*The particle size properties of particles generated using different milling itineraries were characterized.* Equipment used in milling itineraries: drying oven (Memmert, D); microwave oven (Samsung 1000, K); coarse milling with a cutting mill (SM300, Retsch, D); intermediate milling with an impact mill (UPZ, Hosokawa-Alpine, D); fine milling with a vibrating ball-mill (DM1,Sweco, B) and a stirred beads mill (Femag, F). Equipment used in powder characterization: laser diffraction granulometer (Malvern 2000, UK) in dry or wet mode; in the latter mode, an embedded ultrasonic probe (75 W, 0 – 100% tunable) can be used.
Data format	*Raw and analyzed*
Parameters for data collection	Oven or microwave dried samples were milled to reach median particle sizes in between 10-20 µm. Depending on the input size of samples, 3 successive steps of milling (coarse, intermediate, and fine milling) or 1 (fine milling) were applied. The kinetics of particle size reduction were measured by laser granulometry (dry or liquid mode) after periodical sampling. PSD indicators were recorded.
Description of data collection	Data from the milling of 9 different biomass samples (crop and wood by-products) were collected. Table 1: Processing parameters (processing itineraries, machine operating conditions, milling time…) for 9 crop by-products itineraries and 10 wood by-products itineraries. Table 2: Data from granulometric characterization of all ground powders (d10, d50, d90, span, Specific Surface Area). Table 3: The kinetics of particle size reduction for a collection of powder samples (11 for crop by-products and 10 for wood by-products) Table 4: Whole set of full PSD for all samples of Table 3.
Data source location	INRAE, PLANET-IATE, 1208. Agropolymers and Emerging Technologies Facility, *Montpellier FR-34060, France*https://doi.org/10.15454/1.5572338990609338E12
Data accessibility	Data are accessible in a public repository Repository name: INRAE dataverse (https://data.inrae.fr/) Data identification number: 10.15454/S660LH, 10.15454/YZJXET Direct URL to data: https://data.inrae.fr/dataset.xhtml?persistentId=doi:10.15454/S660LH (v9.0) corresponding to wood by-products data, https://data.inrae.fr/dataset.xhtml?persistentId=doi:10.15454/YZJXET (v8.0) corresponding to crop by-products data.

## Value of the Data

• The fine milling of lignocellulosic biomasses is not trivial due to a strong anisotropy and an elastoplasticity behaviour [5,6]. These data obtained with different types of mill and from various biomasses might guide the choice of starting feedstock and processing technologies to better exploit biomass potential according to the targeted applications as petrol replacers.
• The data can benefit to scientists in the sectors of plant and wood products valorization, bio-based energy vectors [7], bio-sourced chemicals [8] and materials [9]. Companies manufacturing plant and wood based powders for food/non-food applications and mill suppliers may be interested. The information from the dataset can help to optimize the processes and the quality of the end-products from lignocellulosic biomass.
• The set of data can allow classifying biomasses according to the milling type and the milling time required to reach a targeted mean particle size. As milling equipment used are batch mills, the milling time is directly related to the milling energy. The dataset can be used to compare new biomass samples and other types of mill. It can also serve as a basis for understanding the mechanism of comminution.
• According to the nature of the starting biomass material and the type of mechanical load applied in the mill, variability in particle characteristics can be introduced [10]. This variability influences the properties of the products from lignocellulosic biomass. These data can be used either to produce tailor-made powders for targeted applications or to better control the quality of the end products.
• The data from the kinetics of particle size reduction can allow determining the minimum energy input to reach an intended particle size with a given milling technology [6].
• These dataset may also open new perspectives for wider uses of biomass in innovative applications, for which advanced granulometric reduction is required (e.g. 3D-printing, smart materials, solid biofuels…) [11], [12], [13].

## 1 Data Description

Data are stored in two datasets (see Data accessibility in the Specification Table above): the first one contains wood by-products data and the second one crop by-products data.

In each dataset, the data are gathered in four files. File Table 1 provides a precise description of milling itineraries associated with produced powder samples. File Table 2 reports the granulometric characteristics of all produced powder samples. The kinetics of particle size reduction for a subset of produced powder samples are gathered in file Table 3. An additional file Table 4 contains the whole set of full particle size distributions of all samples of Table 3. In this Excel file, each sample is stored in a separate thumbnail.

In Table 1, a milling itinerary is described by a set of unit operations and associated with a unique numerical identifier (column *Experience number*). Each unit operation of a given itinerary is described on a separate line and associated with a unique numerical identifier (columns *Experience number* + *Process step number*). Numerical value associated with *Process step number* column indicates temporal order (e,g step 1 is before step 2). In case of parallel unit operations, a dot is used (e,g step 1.1 is parallel to step 1.2). The following information are reported: date; biomass nature and name (Biomass)^1^; processing operation type (Treatment); drying mode/moisture content (Treatment); biomass quantity; equipment name and type (Material); rotation speed; vibration frequency; mass of balls; sieving grid size (sieving size); treatment duration; temperature; output solid quantity (Output solid constituent quantity); output solid yield (Output solid constituent quantity), characterized sample product name (Sample Product1).

In Table 2, are found the data concerning particles and powders characterization: characterized sample product name (Sample Product); biomass name and nature (Biomass); d50 calculation method; d50 in volume; d50 in number; d10 calculation method; d10 in volume; d10 in number; d90 calculation method; d90 in volume; d90 in number; span; specific surface area calculation method; specific surface area value.

Table 3 has the same structure that Table 2 extended by an additional column (Sampling time) indicating the time at which a sample has been extracted during the milling for granulometric characterization. Sample product contains the same names as in Table 2 followed by a label indicating chronological ordering. By example, PB4-T0 to PBA-T7 corresponds to the sample PB4 followed by the sampling time (T0 to T7). PB4 in Table 3 corresponding to the final sampling time is the same sample than in Table 2.

**Fig. 1:**
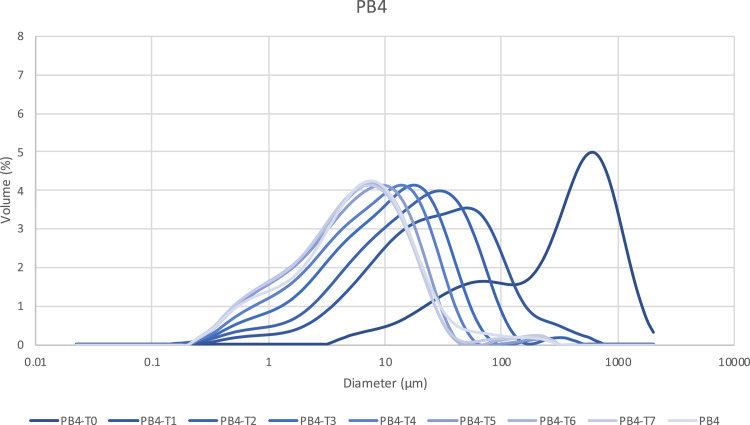
Example of milling kinetics for Pine Bark (PB4) excerpt from Table 4. The sample name (PB4-T0 to PBA-T7) corresponds to the reference of the sample PB4 followed by the sampling time (T0 to T7).

In Table 4, are stored the whole set of full PSD for all samples and associated graphical representations of the evolutions of PSD along the fine milling process of the samples. Fig. 1 provides an example of the milling kinetics of Pine bark (Sample Product PB4) excerpt from Table 4 to reach a median particle size of 5.614 μm. The corresponding milling times are also available in table 4.

These data are stored in INRAE dataverse and replicated in @Web data warehouse [4] (https://www6.inra.fr/cati-icat-atweb/) in which the data structuration and vocabulary standardization are controlled by BIOREFINERY ontology [[3], [4]]. BIOREFINERY ontology is available in INRAE dataverse (https://doi.org/10.15454/X2MOWO, version 2.0) and Agroportal (http://agroportal.lirmm.fr/ontologies/BIOREFINERY, version 2.1). Tutorials associated with atWeb querying module are available at https://www6.inrae.fr/cati-icat-atweb/Tutorials/Querying-thumbnail.

## 2 Experimental Design, Materials and Methods

The information related to the processing of every specific sample are recorded in the milling itineraries table (Table 1). For coarse milling, the SM 300 cutting mill (Retsch, D) operating at 3000 rpm and equipped with a 2 mm grid was used. For intermediate milling the UPZ impact mill (Hosokawa-Alpine, D) operating at 18000 rpm with a grid of 0.3 mm was used. For fine milling (i) the vibrating ball-mill (Sweco, B) filled with 25 kg of 12 mm diameter balls and 25 kg of cylindrical bodies 12 × 12 mm operated at 25 Hz was used to treat 1 kg lignocellulosic samples in batch, or (ii) the stirred beads mill (Femag, F) filled with 7.5 kg of 6 mm-diameter beads with a rotor speed comprised between 300 and 400 rpm was used to treat 170–200 g lignocellulosic samples in batch. Note that samples were dried before fine milling either in a conventional drying oven (Memmert, D) at 60 °C or in a 100 W microwave oven (Samsung 1000, K) to reach a water content below 6%. Aliquots were sampled at various periods of time during the fine milling process and analyzed by laser granulometry (Malvern 2000, UK) to allow the kinetical description of the particle size reduction process. Five different Standard Operating Procedures described below (SOP 1 to 5) were employed according to the nature and the quantity of the sample available. The particle size distribution (PSD) of the powders was measured in either dry (SOP 1) or liquid way (SOP 2 to 5). The conditions of PSD measurement are stated in Table 1 for each sample. The median particle size d(50), the specific surface area (SSA) and the polydispersity index or span which describe the width of the PSD were also extracted from the PSD, with span=(d(90)-d(10))/(d(50)), where d(90), d(50) and d(10) represent the 90th, 50th and 10th percentile of the particle size distribution, respectively. Some powder samples (milled rice husk) were de-agglomerated before particle size measurement (SOP 5).

*Standard operating procedure 1:* Measurements were realized in dry way, using a vibrating hopper Sirocco 200 settled at 80% maximum vibration and 3 bars air pressure. The size of the powder is determined based on Fraunhofer theory [14].

*Standard operating procedure 2:* Measurements were realized in liquid way. Samples were dispersed in ethanol (96 % v/v) in the Hydro 2000S system with a pomp speed of 3000 rpm. The refractive index of ethanol (1.45) and sawdust (1.53), were used to proceed the data based on the Mie Theory [9]. Measurements were carried out in triplicates with a delay of 3 s between each measurement.

*Standard operating procedure 3:* Measurement were realized in liquid way. Samples were dispersed in a mixture of purified water/ethanol (50% v/v) in the Hydro 2000S system with a pomp speed of 3000 rpm. The refractive index of the solvent mixture (1.345) and sawdust (1.53), were used to proceed the data based on the Mie Theory. Measurements were carried out in triplicates with a delay of 3 s between each measurement.

*Standard operating procedure 4:* Measurement were realized in liquid way. Samples were dispersed in ethanol (96 % v/v) in the Hydro 2000S system with a pomp speed of 3000 rpm. The refractive index of ethanol (1.45) and sawdust (1.53), were used to proceed the data based on the Mie Theory. Measurements were carried out just once during the procedure.

*Standard operating procedure 5:* Measurement were realized in liquid way in ethanol (96 % v/v) in the Hydro 2000S system. An ultrasonic treatment was applied prior to the measurement to deagglomerate the powder. 3 min of sonication using the granulometer internal ultrasonic probe at 100% of its power (75W) was applied to a suspension of approximatively 0.1 g of powder in 200 ml of ethanol. The suspension was then stirred at 3000 rpm during 3 min to remove eventual bubbles prior to the measurement. Measurements were carried out in triplicate with a delay of 20 s between each measurement. The refractive index of ethanol (1.45) and sawdust (1.53), were used to proceed the data based on the Mie Theory.

## Ethics Statement

This work neither involve human subject nor animal experiments.

## Declaration of Competing Interest

The authors declare that they have no known competing financial interests or personal relationships which have, or could be perceived to have, influenced the work reported in this article.

## Footnotes

1 (name in brackets indicates corresponding column header name in the table when it differs)
